# Role of JAK/STAT in Interstitial Lung Diseases; Molecular and Cellular Mechanisms

**DOI:** 10.3390/ijms22126211

**Published:** 2021-06-09

**Authors:** Paula Montero, Javier Milara, Inés Roger, Julio Cortijo

**Affiliations:** 1 Department of Pharmacology, Faculty of Medicine, University of Valencia, 46010 Valencia, Spain; irola3@gmail.com (I.R.); julio.cortijo@uv.es (J.C.); 2 Biomedical Research Networking Centre on Respiratory Diseases (CIBERES), Health Institute Carlos III, 28029 Madrid, Spain; 3 Pharmacy Unit, University General Hospital Consortium of Valencia, 46014 Valencia, Spain; 4 Research and Teaching Unit, University General Hospital Consortium, 46014 Valencia, Spain

**Keywords:** interstitial lung disease (ILD), Janus kinases (JAK), signal transducer and activator of transcription (STAT), idiopathic pulmonary fibrosis (IPF)

## Abstract

Interstitial lung diseases (ILDs) comprise different fibrotic lung disorders characterized by cellular proliferation, interstitial inflammation, and fibrosis. The JAK/STAT molecular pathway is activated under the interaction of a broad number of profibrotic/pro-inflammatory cytokines, such as IL-6, IL-11, and IL-13, among others, which are increased in different ILDs. Similarly, several growth factors over-expressed in ILDs, such as platelet-derived growth factor (PDGF), transforming growth factor β1 (TGF-β1), and fibroblast growth factor (FGF) activate JAK/STAT by canonical or non-canonical pathways, which indicates a predominant role of JAK/STAT in ILDs. Between the different JAK/STAT isoforms, it appears that JAK2/STAT3 are predominant, initiating cellular changes observed in ILDs. This review analyzes the expression and distribution of different JAK/STAT isoforms in ILDs lung tissue and different cell types related to ILDs, such as lung fibroblasts and alveolar epithelial type II cells and analyzes JAK/STAT activation. The effect of JAK/STAT phosphorylation on cellular fibrotic processes, such as proliferation, senescence, autophagy, endoplasmic reticulum stress, or epithelial/fibroblast to mesenchymal transition will be described. The small molecules directed to inhibit JAK/STAT activation were assayed in vitro and in in vivo models of pulmonary fibrosis, and different JAK inhibitors are currently approved for myeloproliferative disorders. Recent evidence indicates that JAK inhibitors or monoclonal antibodies directed to block IL-6 are used as compassionate use to attenuate the excessive inflammation and lung fibrosis related to SARS-CoV-2 virus. These altogether indicate that JAK/STAT pathway is an attractive target to be proven in future clinical trials of lung fibrotic disorders.

## 1. Introduction

Interstitial lung diseases (ILDs) comprise a heterogeneous group of disorders characterized by lung damage as a result of lung fibroblast proliferation, interstitial inflammation, and fibrosis [1]. Apart from the lung interstitium, these diseases can affect airspaces, peripheral airways, and vessels, along with their respective epithelial and endothelial linings [2]. In its broader classification, ILDs can be distinguished between those with known and unknown cause. Drug and radiation-induced lung disorders, pulmonary hemorrhages syndrome, chronic hypersensitivity pneumonitis, pulmonary sarcoidosis, or ILDs associated with autoimmune diseases like systemic sclerosis (SS) and rheumatoid arthritis (RA) are classified as interstitial lung diseases with known etiology [3]. The American Thoracic Society (ATS) and European Respiratory Society (ERS) subdivides the unknown etiology ILDs into three groups in which idiopathic interstitial pneumonias are the major ones [2,4]. Inside the major group (Table 1), there are chronic fibrosing disorders, such as idiopathic pulmonary fibrosis (IPF) and idiopathic nonspecific interstitial pneumonia (NSIP), smoking-related disorders, such as respiratory bronchiolitis–interstitial lung disease (RB–ILD), desquamative interstitial pneumonia (DIP), and acute/subacute idiopathic interstitial pneumonias, such as cryptogenic organizing pneumonia (COP) and acute interstitial pneumonia (AIP) [5]. The ATS/ERS also accounts for two rare conditions, lymphoid interstitial pneumonia (LIP) and idiopathic pleuroparenchymal fibroelastosis (PPFE).

ILDs manifestations are characterized by different grades of inflammation or fibrosis in the lung parenchyma. In inflammation disease, the histology is characterized by organizing pneumonia or non-specific interstitial pneumonia, while fibrosis dominant disease is characterized by the usual interstitial pneumonia (UIP) pattern, characterized by fibroblastic foci and mild to moderate inflammation [11]. From all the conditions listed above, IPF was described the most, being the most common progressive, fibrotic disease [1]. Table 1 shows the main histologic and radiologic differences between the major idiopathic interstitial pneumonias. However, managing ILDs is a challenge for clinicians. Pathologic and clinical similarities amongst ILDs make diagnostic difficult. Therefore, multidisciplinary evaluation is usually recommended, including the discussion of clinical, radiological, and pathological data from lung biopsy if required [12].

Janus kinases (JAK) are a group of intracellular tyrosine kinases (JAK1, JAK2, JAK3, TYK2) that are crucial in signal transduction initiated by a wide range of membrane receptors [13]. Signal transducer and activator of transcription (STAT) comprises a family of 7 members (STAT1, STAT2, STAT3, STAT4, STAT5a, STAT5b, STAT6), which function as transcription factors [14]. The JAK/STAT signaling pathway controls multiple cellular processes that are essential for cell homeostasis. Alterations in this axis play a role in cancer progression [15] and inflammatory and autoimmune diseases [16,17,18]. Between different JAK/STAT isoforms, it appears that JAK2/STAT3 are predominant, initiating cellular changes observed in ILDs. This review analyzes the expression and distribution of different JAK/STAT isoforms, the implications of its downstream pathways in the cellular processes related to ILDs, and its therapeutic management. ILDs caused by COVID-19 infection, their relationship with JAK/STAT, and therapeutic strategies will be analyzed.

## 2. JAK/STAT Activation Mechanisms

**Figure 1:**
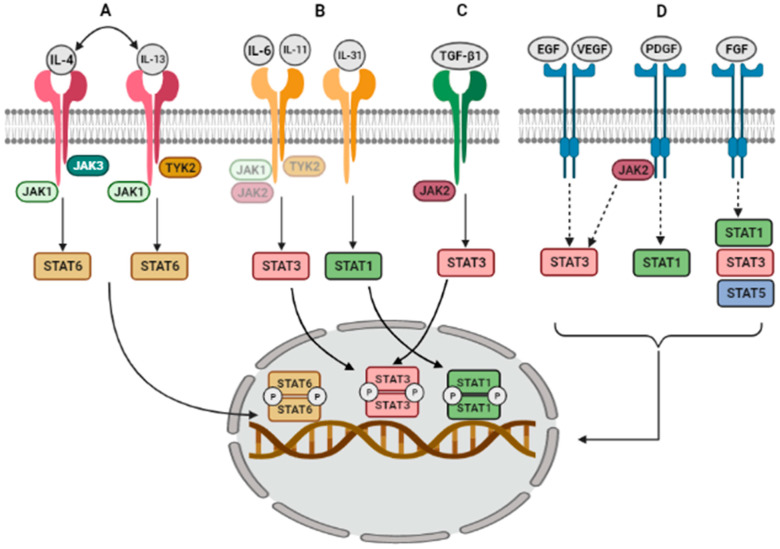
JAK/STAT activation and downstream signaling pathways in ILDs. (**A**) IL-4 binds to the type-I IL-4 receptor, constituted by the IL-4 receptor α chain (IL-4Rα) and the gamma chain (γc). Additionally, both IL-4 and IL-13 bind to the type II receptor made up of IL-4Rα/IL-13Rα1. Stimulation of these receptors activates IL-4Rα and associated JAK1 to phosphorylate STAT6 monomers, which then homodimerize and translocate to the nucleus when it activates fibrotic responses. (**B**) IL-6 and IL11 activate the receptor gp130. This receptor has shown binding to JAK1, JAK2, and TYK2 in other diseases; however, in ILDs, is not elucidated, which JAK induces the phosphorylation of STAT3. The same happens after gp130 stimulation by IL-31, which leads to STAT1 activation. Then, both STAT3 and STAT1 travel dimerized to the nucleus. (**C**) Non-canonical activation of TGF-β1 signaling pathway starts with its binding to the TGF-β1 receptor, and then the phosphorylation of JAK2 occurs, leading to STAT3 activation and homodimerization. (**D**) Indirect evidence of JAK/STAT pathway activation by growth factors. Here, there are represented the different JAKs and STATS found activated after stimulation of each receptor with epidermal growth factor (EGF), vascular endothelial growth factor (VEGF), platelet-derived growth factor (PDGF), and fibroblast growth factor (FGF) in other diseases different to ILD. The image was created with BioRender.

Figure 1 shows the activation mechanisms in JAK/STAT pathway. It starts with the binding of a ligand, usually a cytokine, growth factor, or interferon, to its receptor inducing its dimerization. Then, receptor-associated JAKs are activated and phosphorylate the tyrosine residues of the intracellular tail of their receptors. These phosphorylations serve as docking sites for signal transducers and activators of transcription (STATs) and bind to them through their SH2 domain. Thus, the STATs become activated by JAKs and translocate to the nucleus to regulate gene expression. However, some variations of this signaling pathway were described as non-canonical. This can happen whenever a receptor dimerizes in the absence of the ligand, when STATs dimers in the absence of the activating phosphorylation by a conformational change, or when latent STATs remain in a state of nuclear import and export [19]. Table 2 summarizes the ligands that activate the different JAK/STAT proteins in ILDs and in their corresponding animal models, which will be described below.

### 2.1. Activation by Interleukins

Several cytokines, which activate a JAK/STAT pathway, like IL-4, IL-13, IL-6, IL-11, and IL-31, are implicated in the pathogenicity of ILDs.

IL-4 and IL-13 are related to ILDs [42] and are required for the maintenance of pulmonary fibrosis [43], modulating the abnormal activation of lung fibroblast [44]. Both cytokines share their receptor structure and common downstream signaling. From the two types of receptors, subunit 1 binds to TYK2, activating JAK1, STAT3, and STAT6 [45]. In AECs from both a BLM-induced fibrosis mouse model and pulmonary fibrosis patients, IL-4 and IL-13 mediate their fibrotic effect by binding to the receptor and activating JAK1. In turn, JAK1 phosphorylates STAT6 prompting STAT6 to homodimerize and translocate to the nucleus [20,21].

Another shared signaling pathway involves the gp130/IL-6 receptor family, activated by IL-6, IL-11, and IL-31, and mediated by STAT3 [46]. IL-6 mediates different inflammatory processes in the lungs, and its dysregulated expression was implicated in the pathogenesis of interstitial pneumonias and IPF [47,48]. In a BLM-induced mouse model, IPF fibroblasts, and normal lung fibroblasts, STAT3 is activated after IL-6 binding to the receptor gp130 [22,23,24,49]. IL-11 is upregulated in scleroderma-associated interstitial lung disease and IPF patients [50]. Stimulation of lung fibroblasts with IL-11 induces STAT3 phosphorylation [27]. IL-11 also signals its fibrotic properties through STAT3 in BLM-induced fibrosis mouse model [25,26]. Regarding gp130 activation by IL-31, there is not much evidence of its relationship with ILDs, but one study in a mouse pulmonary fibrosis model found that IL-31 signals through STAT1 activation, and may constitute a possible pathway of mouse pulmonary fibrosis [28].

### 2.2. Activation by Growth Factors

JAK/STAT axis is also activated by growth factors. One of them is transforming growth factor β1 (TGF-β1), the principal pro-fibrotic factor promoting fibroblasts to myofibroblasts differentiation in ILDs [51]. Usually, its activation leads to the phosphorylation of the signal transducer proteins SMAD2/3, which translocate to the nucleus to activate pro-fibrotic genes. However, an alternative TGF-β1 activation pathway has also been described. This alternative pathway is SMAD-independent and involves STAT3 activation (Figure 1). In AECs and fibroblasts, TGF-β1 activates STAT3 [30]. Other studies in human fibroblasts and mouse models of systemic sclerosis describe JAK2 activation by TGF-β1, which in turn phosphorylates STAT3 and leads to its nuclear translocation [29].

In IPF, our group has described that TGF-β1 activates the pathway by JAK2 phosphorylation. Moreover, p-JAK2 was found in the nucleus of fibrotic areas, a fact that implies that it may act independently of STAT3 [31]. The same signaling is observed in systemic sclerosis (SS), where TGF-β1 induces JAK2 phosphorylation, which then interacts with phosphorylated STAT3 to induce fibrotic responses. Interestingly, JAK2 may not only be a downstream target of TGF-β1 in fibroblasts but also performs positive feedback, amplifying TGF-β1 signaling by stimulating more TGF-β1 expression [32].

The JAK/STAT pathway and its different activators are widely described in cancer, but its implication in ILDs is still poorly understood. Therefore, some studies analyze the implication of JAK/STAT activation molecules in ILDs, but do not describe the mechanism, nor which JAKs and STATs are implicated in that activation. This happens with epidermal growth factor (EGF), platelet-derived growth factor (PDGF), fibroblast growth factor (FGF), and vascular endothelial growth factor (VEGF).

EGF is upregulated in IPF, COP, and fibrotic NSIP [33], and is linked to STAT3 activation in cancer progression [34]. PDGF is increased in alveolar macrophages in patients with ILD [35] and bronchoalveolar lavage fluid (BALF) and lung samples from patients with IPF [36]. PDGF activates STAT1 signaling in primary lung fibroblasts [37] and JAK2/STAT3 in atrial fibroblasts in dogs with heart failure [52]. FGF is also increased in the lung of progressive fibrosing-ILD [53] and activated FGF-receptor phosphorylates STAT1, STAT3, and STAT5 [38,39,54]. However, none of the growth factors listed above were proven to signal through JAK/STAT in ILDs. VEGF shows conflicting results as to whether it is a contributory or protective factor. Recent evidence shows that VEGF constitutes an exhaled biomarker in IPF, with significant correlations with the clinical manifestations [55], and patients with idiopathic interstitial pneumonia have higher VEGF levels in plasma [56]. VEGF is associated with STAT3 in primary astrocytes when ionizing radiation-induced overexpression of VEGF leads to STAT3 activation [40]. Moreover, STAT3 has a binding site on the VEGF promoter and can regulate VEGF expression [41]. Again, no direct associations between VEGF and STATs were described in ILDs.

## 3. JAK/STAT Lung Expression and Distribution

Amongst the different ILDs, the expression and distribution studies that analyze JAK and STAT protein families focus mainly on IPF and describe primarily JAK1, JAK2, and STAT1, STAT3. Table 3 shows a summary of the studies that analyze JAKs and STATs expression and distribution in animal models and patients.

JAK1 is overexpressed in lung tissue and localized in inflammatory and epithelial cells of bleomycin (BLM)-induced fibrosis mouse model. Its active form is also increased in the lungs from this mouse model [57,58].

Our group described that JAK2 is overexpressed in a BLM-induced fibrosis rat model, as well as its phosphorylated form. In IPF patients, JAK2 is distributed in hyperplastic alveolar epithelial type II cells (ATII), fibroblasts, and tunica intima, and media of small pulmonary arteries. p-JAK2 is detected in lung tissue from IPF patients, being both JAK2 and p-JAK2 expression increased in lung tissue and pulmonary arteries [31,59].

Regarding the STAT family, STAT1 expression and distribution are mostly analyzed in animal models. In BLM-induced fibrosis mouse models, STAT1 is found in inflammatory and epithelial cells, whereas in rats is appreciable in alveolar macrophages [28,57,60,61]. Further, p-STAT1 was detected in lung tissue from BLM-induced fibrosis mice [58].

STAT3 is the isoform that gathers most evidence related to ILDs. Its distribution is higher in alveolar macrophages, endothelial cells, and neutrophils in BLM-induced fibrosis mouse model. Its phosphorylated form is also present in myofibroblasts and alveolar macrophages. In the same model in rats, STAT3 is found in fibrotic cells and p-STAT3 in the nuclei of such fibrotic cells. Gene and protein STAT3 expression is elevated in this mouse model, as well as p-STAT3 protein expression in lung tissue. Similarly, p-STAT3 is increased in lung tissue from BLM-induced fibrosis in rats [31,57,61,62]. In IPF patients, STAT3 is present in the tunica intima and media of small pulmonary arteries, hyperplastic alveolar cells, and fibroblasts. Its active form was detected in areas of dense fibrosis in IPF patients, more accurately in the nucleus of pulmonary artery cells, in myofibroblasts, and the nuclei of alveolar epithelial cells (AECs) [23,31,49,59,61].

Interestingly, there is one study showing evidence for increased expression of STAT6 in IPF, but no further studies have described this isoform expression or distribution in ILDs [20].

Although the expression and distribution of the JAK/STAT axis are mostly analyzed in IPF, some studies describe it in other ILDs. For instance, gene expression bioinformatic studies showed that STAT1 is upregulated in blood and lung samples from sarcoidosis patients [63,64]. TYK2 is upregulated in BALF from progressive pulmonary sarcoidosis patients [65]. Finally, in a DIP mouse model, there is increased p-STAT5 protein expression in alveolar macrophages [66].

**Table 3:** Summary of JAK/STAT expression and distribution studies.

JAK/STAT	Study Subject/ILD	JAK/STAT Lung Distribution	JAK/STAT mRNA and Protein Expression	Reference
JAK 1	BLM-induced IPF mouse model	Inflammatory and epithelial cells	Increased mRNAexpression in lung tissue	[57]
p-JAK1	BLM-induced IPF mouse model	Lung tissue		[58]
JAK2	BLM-induced IPF rat model	Fibrotic cells		[31]
	IPF patients	Hyperplastic alveolar cells and fibroblasts	Increased mRNA and protein expression in lung tissue	[31]
	IPF + pulmonary hypertension patients	Tunica intima and media of small pulmonary arteries	Increased mRNA and protein expression in pulmonary arteries	[59]
p-JAK2	BLM-induced IPF rat model	Nuclei of fibrotic cells	Increased protein expression in lung tissue	[31]
TYK2	Progressive pulmonary sarcoidosis patients		Increased mRNA expression in bronchoalveolar lavage	[65]
STAT1	BLM-induced IPF mouse model	Inflammatory and epithelial cells	Increased mRNAexpression in lung tissue	[28,57,60]
	BLM-induced IPF rat model	Alveolar macrophages		[61]
	Sarcoidosis patients		Increased expression in blood and lung samples	[63,64]
p-STAT1	BLM-induced IPF mouse model	Lung tissue		[58]
STAT 3	BLM-induced IPF mouse model	Alveolar macrophages, endothelial cells, and neutrophils	Increased mRNA and proteinexpression in lung tissue	[30,57]
	BLM-induced IPF rat model	Fibrotic cells	Increased protein expression in lung tissue	[31]
	IPF patients	Hyperplastic alveolar cells and fibroblasts	Increased mRNA and protein expression in lung tissue	[31]
	IPF+ pulmonary hypertension patients	Tunica intima and media of small pulmonary arteries	Increased mRNA and protein expression in pulmonary arteries	[59]
p-STAT3	BLM-induced IPF mouse model	Myofibroblasts and alveolar macrophages	Increased protein expression in lung tissue	[30,62,67]
	BLM-induced IPF rat model	Fibrotic cells and nuclei of fibrotic cells	Increased protein expression in lung tissue	[31]
	IPF patients	Areas of dense fibrosis, parenchymal cells adjacent to collagenous foci, (epithelial and hematopoietic origin), alveolar macrophages, myofibroblasts and nuclei of AECs	Increased protein expression in lung tissue and isolated IPF-lung-fibroblasts	[23,30,49,67]
	IPF+ pulmonary hypertension Patients	Nucleus of pulmonary artery cells, fibroblasts, and AECs	Increased protein expression in pulmonary arteries	[59]
p-STAT5	DIP mouse model		Increased p-STAT5 protein expression in alveolar macrophages	[66]
STAT 6	BLM-induced IPF mouse model		Increased mRNA expression	[20]

Abbreviations: idiopathic pulmonary fibrosis (IPF), bleomycin (BLM), alveolar epithelial cells (AECs).

## 4. Genetic Alterations

Apart from its aberrant expression, another way that relates JAKs and STATs with ILDs is the different genetic alterations that cause disease. The most common are known as germline gain-of-function (GOF) mutations. STAT3 GOF mutations lead to an increased transcriptional activity that causes autoimmunity and lymphoproliferation. These mutations have also been associated with the development of different ILD, such as LIP [68,69,70,71], DIP [72], UIP, and COP [73,74]. However, the mechanism by which STAT3 GOF causes ILD is not currently elucidated [75]. Another alteration described is the deficiency in STAT5B. Homozygous recessive mutations in STAT5B in humans result in growth hormone insensitivity (GHI) and primary immunodeficiency. This deficiency usually causes lymphocytic interstitial pneumonia (LIP) [76,77,78]. Both STAT3 GOF and STAT5B deficiency result in impairment of T_reg_ function, which explains the autoimmune manifestations, but how autoimmunity affects the development of ILD needs further study [79]. Intriguingly, despite all JAK/STAT genetic alterations lead to a bad overcome in ILDs, the STAT4 rs7574865 T allele may be protective against the development of lung fibrosis in SS patients [80].

## 5. Cellular Processes Activated by JAK/STAT

**Figure 2:**
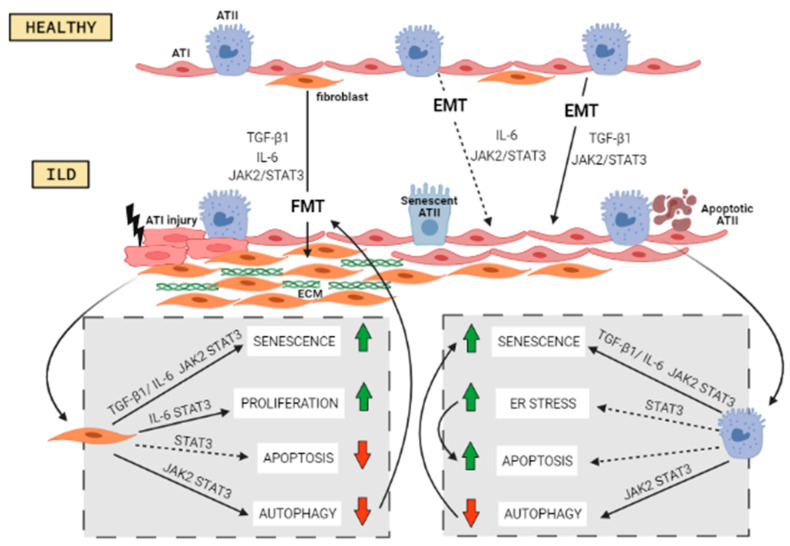
Cellular processes implicated in the pathogenesis of ILDs, and their relationship with JAK/STAT signaling pathway. Abbreviations: Alveolar type II cell (ATII), alveolar type I cell (ATI), epithelial to mesenchymal transition (EMT), fibroblast to mesenchymal transition (FMT), extracellular matrix (ECM). The image was created with BioRender.

Figure 2 shows the different cellular processes implicated in the pathogenesis of ILDs, and their relationship with JAK/STAT signaling pathway, which will be discussed below.

### 5.1. Fibroblasts to Mesenchymal Transition

Fibroblast to mesenchymal transition (FMT) is a cellular process in which fibroblasts lose their differentiation and acquire the mesenchymal phenotype of myofibroblasts, characterized by the expression of alpha smooth muscle actin (α-SMA) and an aberrant expression of extracellular matrix components (ECM). In other cellular types, such as hepatocytes, IL-6 induces its transition towards myofibroblast through STAT3 signaling [81]. Further, STAT3 regulates IL-6–mediated and TGF-β1–mediated myofibroblast differentiation in murine lung fibroblasts [30], and inhibition of STAT3 was found to reduce TGFβ1-induced FMT in fibroblasts, and ameliorate skin fibrosis in two mouse models of systemic sclerosis [82]. Pechkovsky also pointed out that STAT3 might be involved in the regulation of collagen I secretion by lung fibroblasts in IPF and that enhanced expression of STAT3 in IPF fibroblasts might be responsible for their fibrogenic phenotype [23]. Consistent with these findings, our group observed that both p-STAT3 p-JAK2 inhibition in lung fibroblasts from IPF patients partially reduced FMT induced by TGF-β1 and IL-6/IL-13 [31]. These findings indicate that JAK2/STAT3 might be driving FMT in IPF, although there is no evidence in other ILDs.

### 5.2. Epithelial to Mesenchymal Transition

Another cellular transformation found in fibrotic diseases is epithelial to mesenchymal transition (EMT), which allows epithelial cells to convert into motile mesenchymal cells [83]. This transition is implicated in the regulation of metastasis and cancer progression and is driven by the IL-6/JAK2/STAT3 pathway in lung and ovarian carcinomas [84,85]. In HepG2 cells, this pathway upregulates the expression of Twist, a key transcription factor of EMT [86]. Moreover, IL-6 secreted by activated fibroblasts in cancer stroma induces EMT of gastric cancer cells via JAK2/STAT3 [87]. In peritoneal fibrosis, IL-6/JAK2/STAT3 acts as a pro-EMT pathway [88].

In pulmonary fibrosis, EMT was described as a source of myofibroblasts in in vitro studies, animal models, and patients [89]. In IPF, EMT in ATII cells contributes to the pathology of the disease [90,91]. Nonetheless, there are no studies that describe STAT3 as a mediator of the EMT process in ILDs, although our group observed that TGF-β1 induced EMT in ATII cells was attenuated by dual p-JAK2/p- STAT3 inhibition, showing that this regulator might be directly involved [31].

### 5.3. Senescence

Cellular senescence is a stable cell cycle arrest that maintains cells viable and metabolically active. It is a physiological response to prevent genomic instability and accumulation of DNA damage [92]. This process is implicated in IPF pathogenesis. Epithelial cells and fibroblasts from IPF lung tissue have increased senescence biomarkers p16 and p21, and senescence-associated β-galactosidase activity (SA-β-gal) [93], and show elevated senescence-associated secretory phenotype (SASP) components [94,95]. This phenotype contributes to cytokine-stimulated gene transcription, and one cytokine altered is IL-6 signaling through STAT3/JAK1 and JAK2 [96,97]. In cancer, IL-6 and STAT3 are related to the induction of senescence [98] and in lung fibroblasts, STAT3 is an important factor driving oxidant-induced senescence [99]. Waters and colleagues showed that targeting STAT3, protected against lung fibroblast cell senescence, similarly to our results, in which the senescence responses induced by TGF-β1 in fibroblasts and A549 alveolar type II cells were suppressed by dual si-RNA-JAK2/STAT3 inhibition [31]. Taken together, although there is no research in the direct association of STAT3 mediated senescence in ILDs, all evidence suggests its possible implication.

### 5.4. Apoptosis and Proliferation

As a result of senescence, apoptosis and proliferation are reduced [94]. However, opposite results are described in both cell processes related to ILDs.

Apoptosis is a process of programmed cell death that regulates cell number to maintain the homeostasis of many adult tissues. ATII cells in lung from IPF patients actively undergo programmed cell death [100] as well as in murine models of BLM-induced pulmonary fibrosis [101]. Indeed, apoptosis of AECs is considered a key incident initiating the pathogenesis of IPF [102]. On the contrary, myofibroblasts in IPF appear to be apoptosis-resistant [103]. There is evidence for STAT3 role in the resistance of fibroblasts to ultraviolet- and interferon (IFN)-γ–induced apoptosis [104]. Moreover, STAT3 overexpression in human lung fibroblasts induces resistance to FasL-induced apoptosis [46]. Moodley et al. could associate the implication of STAT3 in IPF-fibroblast resistance to apoptosis, demonstrating that treatment of IPF fibroblasts with IL-6 conferred resistance to FasL-induced apoptosis, an effect mediated by STAT3 [22]. However, further studies are needed to designate STAT3 as a mediator driving apoptosis in ILDs. Regarding proliferation, this process is also a hallmark of fibrotic-associated ILDs, and it is associated with the IL-6/gp130 signaling pathway. In lung fibroblasts derived from IPF patients, stimulation with IL-6 induced proliferation, and a transient STAT3 activation, while in normal fibroblasts, IL-6 stimulation reduced proliferation through STAT3 signaling [24].

### 5.5. Endoplasmic Reticulum Stress

The endoplasmic reticulum (ER) is responsible for the processing of proteins and mediates their folding, assembly, trading, and degradation. The ER is regulated by different elements including protein load, cell metabolism, redox balance, and calcium homeostasis. Any alterations in such factors can induce ER stress [97]. When the protein folding capacity of the ER is altered, the unfolded protein response (UPR) activates to restore homeostasis. When the UPR is prolonged or severe, cells become dysfunctional and undergo apoptotic death [105]. Several reports have suggested the possible implication of ER stress and UPR in ILDs and lung fibrosis. In lung fibrosis, the UPR process is activated in ATII cells [106,107]. Studies on IPF lung samples show increased ER stress and UPR activation-associated proteins [108] and both processes are found in AECs from patients with familial and sporadic IPF [108]. There is also evidence showing that ER stress in ATII could induce the execution of the intrinsic apoptotic pathway [109]. While there is evidence that ER stress drives pathogenesis pathways in ILDs, there are no studies on the role of JAK/STAT. However, STAT3 activation was related to ER stress in other diseases: activation of the ER stress by viruses, such as hepatitis C and RNA-containing viruses, was shown to activate STAT3 through reactive oxygen species (ROS) production [110]. Furthermore, during the acute-phase response in hepatocytes, ER stress is induced in a STAT3-dependent way [111]. Finally, in response to ER stress the PERK/JAK1/STAT3 pathway is activated in primary astrocytes [112]. Overall, these findings indicate the implication of STAT3 in ER stress, but how STAT3 is activated in response to ER stress specifically in ILDs needs further investigation.

### 5.6. Autophagy

Autophagy is a process in which the cell sequestrates dysfunctional cellular cytoplasmic components into vesicles to deliver them to the lysosome for destruction in order to maintain cell survival [113]. When the process gets uncontrolled, it can lead to apoptosis. Autophagy is not induced in pulmonary fibrosis and may represent a mechanism for the promotion of fibrogenesis in IPF due to decreased autophagic activity in lung tissues from IPF patients [114]. Normal fibroblasts activate autophagy in response to stress caused by collagen accumulation. On the contrary, myofibroblasts derived from IPF patients have lower rates of autophagy under the same conditions [115]. In AECs from IPF patients, there is insufficient autophagy, which may lead to epithelial cell senescence, whereas in fibroblasts, lowered autophagy may induce differentiation into myofibroblasts [116]. Further, in BALF cells derived from patients with IPF and RA-ILD, gene expression analysis of autophagy markers showed similar expressions, which indicates that autophagy might be impaired in the same way as IPF in other ILDs [117].

In cancer, STAT3 activates genes of anti-autophagy proteins like BCL2, BCL2L1, and MCL1 [118], which disrupt the formation of the complex BECN1/PIK3C3, essential for autophagy [98]. Both nuclear and cytoplasmic STAT3s were found to contribute to the signaling of autophagy [119]. While the direct contribution of STAT in autophagy in ILDs has not been described, our group found that pharmacological inhibition of JAK2/STAT3 increased autophagy in the lungs of BLM-induced fibrosis in rats [31], which evidences a possible role for this pathway in autophagy in ILDs. However, more studies are still necessary to clarify this role.

## 6. Targeting JAK/STAT for ILDs Treatment

Regarding all evidence suggesting the implication of JAK/STAT signaling pathways in ILDs, it is not surprising that inhibitors targeting JAK/STAT were proposed to treat these diseases. Most of the evidence for the use of JAK/STAT inhibitors in ILDs is described in ILDs associated with another disease, mostly autoimmune, but there are a lack of studies on idiopathic ILDs. Tocilizumab, a monoclonal antibody that competitively inhibits the binding of IL-6 to its receptor [120], is a therapeutic option that acts as an indirect JAK/STAT inhibitor by impeding IL-6/JAK/STAT signaling. Tocilizumab was studied in patients with early and progressive SS-ILD [121]. Aside from tocilizumab, therapeutic options other than JAK/STAT inhibitors are beyond this review.

### 6.1. JAK Inhibitors

For autoimmune and malignant diseases, several JAK inhibitors were developed. The first-generation pan-JAK inhibitors include tofacitinib, ruxolitinib, and baricitinib and are the most described ones for ILDs. Second generation inhibitors are in development and block JAKs with more specificity than first generation inhibitors [122].

#### 6.1.1. Tofacitinib

Tofacitinib was approved for the treatment of RA and is a small-molecule JAK1/JAK3, and to a lesser extent, JAK2/TYK2 inhibitor [123]. The safety and effectiveness of tofacitinib were proven in phase II and III clinical trials for RA [124,125]. It was assessed, as well, in clinical trials in psoriasis, psoriatic arthritis, and inflammatory bowel disease [18]. In vitro and in vivo studies investigated tofacitinib in ILDs. For instance, the pretreatment with tofacitinib, abrogated fibrotic responses induced by IL-6 in normal skin fibroblasts in vitro, although it was not effective when added after the stimulus. Likewise, Tofacitinib acted as a fibrosis preventive agent in a BLM-induced fibrosis mouse model, but failed to act as a therapeutic drug [126]. However, in SKG mice, tofacitinib suppressed the progression of RA-ILD by facilitating the expansion of myeloid-derived suppressor cells in the lungs [127], and in the SS-associated ILD HOCl mouse model, it ameliorated the pro-fibrotic and pro-inflammatory markers [128]. It appears that controversial results are raised regarding the effectiveness of this drug in vivo, may be due to the different ILD models used. In patients, a concise report showed that combination therapy with tofacitinib for refractory anti-MDA5-associated ILD improved the survival rate. However, the number of cases in this study was not enough to reach any conclusions on the efficacy of this therapy [129]. Another single-case report corroborated the efficacy and safety of tofacitinib in combination with nintedanib in the management of an aggressive ILD with poor prognosis [130]. Further, a study showed that tofacitinib was effective in patients with STAT3 GOF causing ILD [73]. Despite this, there is still a lack of evidence for the proposal of tofacitinib as ILDs treatment. However, the recent clinical trial PULMORA (NCT04311567), in recruiting status, will assess the effectiveness of tofacitinib for subclinical and clinical ILD in patients with early RA and might shed some light on this matter.

#### 6.1.2. Ruxolitinib

Ruxolitinib is another JAK1 and JAK2 inhibitor, with moderate activity against TYK2 [131], approved for the treatment of intermediate and high-risk myelofibrosis [123]. In this disease, ruxolitinib inhibits both the wild type and the mutated allele JAK2V617F [132,133]. It was also studied in psoriasis and RA [134]. The effects of ruxolitinib on macrophages were analyzed in an SS-associated ILD mouse model and it ameliorated the pro-fibrotic and pro-inflammatory markers [128]. Moreover, treatment with ruxolitinib in a BLM-induced fibrosis mouse model ameliorated the fibrotic lesion, and reduced levels of fibrotic molecular markers [135]. Although there are no clinical trials analyzing ruxolitinib in ILDs, some case reports were studied. Firstly, ruxolitinib was proven effective in patients with ILD caused by STAT3 GOF that had previously been treated with tocilizumab monotherapy with no improvement reported [73]. Secondly, a case reported the effectiveness and safety of ruxolitinib in one patient with refractory systemic idiopathic juvenile arthritis-associated ILD, which suggests the use of ruxolitinib as a valid therapeutic option for ILDs, although more studies are needed [136].

#### 6.1.3. Baricitinib

Baricitinib is a JAK1/JAK2 inhibitor that was approved for the treatment of RA [137]. Nonetheless, it was not evaluated for the treatment of ILDs. The only evidence remains in two studies. One is a case report of an ILD associated with STING-vasculopathy. This patient failed to respond to ruxolitinib and had to stop treatment due to severe side effects. However, he showed improvement in clinical manifestations when treated with baricitinib [138]. Moreover, baricitinib was analyzed in patients with RA-ILD and promoted the reduction of lung fibrosis and inflammation biomarkers [139].

### 6.2. STAT Inhibitors

Regarding STAT inhibitors, its development raises some selectivity issues. STATs share downstream cascades, and blocking one of them might not stop the signaling pathway activation because another STAT might compensate for the inhibited one and will induce a response [18]. Therefore, there are no clinical trials that study STAT inhibition in ILD patients, but a few STAT3 small-molecule inhibitors were used in vitro and in animal models. In human lung fibroblasts, the nuclear and mitochondrial STAT3 inhibitor, STA-21 attenuates the secretory and senescent profile of these cells [99]. C-188-9 inhibits STAT3 and promotes the decrease of fibrotic markers in the BLM-induced fibrosis mouse model, as well as treatment of primary murine lung fibroblasts with C-188-9 attenuates FMT induced by TGF-β1 [30]. Similarly, the dual p-JAK2/p-STAT3 inhibition with JSI-124 reduces FMT and EMT in lung fibroblast and ATII cells, respectively, and the same inhibitor in BLM-induced fibrosis in rats reduces several markers of fibrosis [31].

## 7. JAK/STAT and COVID-19

### 7.1. Interstitial Lung Disease and COVID-19

Since December 2019, coronavirus disease 2019 (COVID-19) has supposed a serious worldwide health concern. The virus SARS-CoV-2 binds to the surface protein angiotensin-converting enzyme II (ACE2) in ATII cells to infect the lung [140]. After infection, ATII cells and alveolar macrophages trigger an inflammatory response that releases cytokines and chemokines and induces the recruitment of natural killer cells (NK), T-lymphocytes, blood-borne macrophages, and neutrophils, which aggravate the inflammatory response causing the cytokine storm [141]. This storm is characterized by the secretion of acute phase response cytokines IL-6, TNF-α, IL-1β, and then type-1 interferons differentiate NK cells leading to the activation of the adaptive immune response [142]. IL-6 induces endothelial activation, inflammatory cell migration and macrophage activation, which in turn aggravates the inflammatory damage [143]. Acute respiratory distress syndrome (ARDS) can occur as a result of the aforementioned acute systemic inflammatory response [144].

The inflammation stage of COVID-19 shares some similarities with interstitial pneumonia seen in patients with ILD. In fact, it has already been reported that ILD is developed as an important consequence after COVID-19 infection [145,146]. Similarities amongst the histologic and radiologic findings of COVID-19 and ILD can be found [147]. The most common radiological pattern in COVID-19 is bilateral ground-glass opacification with or without consolidation in a subpleural distribution [147]. The histologic findings include diffuse alveolar damage (DAD), desquamation, interstitial fibrosis, and microcystic honeycombing. As previously showed in Table 1, these characteristics are shared with those seen in ILDs [148].

COVID-19 patients also show fibrotic manifestations as a result of lung injury [149,150], and some of the molecular markers seen in COVID-19, are increased in IPF patients, which implies that pulmonary fibrosis has a role in the development of COVID-19 [151]. In IPF pathogenesis, lung damage induces an aberrant wound healing response that leads to the accumulation of fibrotic tissue. Indeed, it can be speculated that pulmonary injuries caused by COVID-19 in severe patients might progress to pulmonary fibrosis in the future [152]. As the pandemic is still in course, and not all data are available, we can rely on results from previous coronavirus outbreaks, like severe acute respiratory syndrome (SARS) and Middle East respiratory syndrome (MERS), to predict how COVID-19-induced fibrosis will affect life quality of disease survivors. Some follow-up studies concluded that fibrosis appears as a common sequelae in severe SARS patients [153,154,155] and in a follow-up study of 36 patients surviving MERS, 33% had pulmonary fibrosis [156]. These data suggest that pulmonary fibrosis prevalence will increase after the COVID-19 pandemic.

### 7.2. The JAK/STAT Pathway in COVID-19

**Figure 3:**
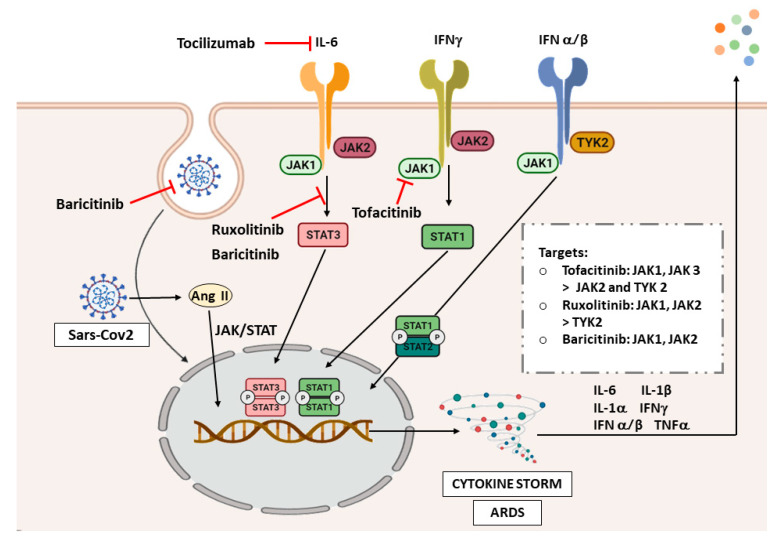
JAK/STAT inhibitors in COVID-19. Abbreviations: interferon (IFN), angiotensin II (Ang II), acute respiratory distress syndrome (ARDS). The image was created with BioRender.

Due to the critical role of inflammation in the pathogenesis of COVID-19, and the histological and radiological similarities with ILDs, we will analyze the possible implication of JAK/STAT signaling in COVID-19 (Figure 3). In the inflammatory response, IFN-γ signals via JAK1 and JAK2 which in turn activate STAT1 [157]. STAT3 contributes to the cytokine storm due to its activation by IL-6 after binding to its receptor gp130 [141]. Another relationship is based on the shedding of ACE2 from the cell surface after endocytosis, a fact that increases the levels of angiotensin II (Ang II) resulting in the development of ARDS. The effect of Ang II is mediated through the JAK-STAT signaling pathway [158] (Figure 3). What is more, a computational method predicted that miRNAs encoded by SARS-CoV-2 target genes in JAK/STAT signaling pathways [159]. Finally, another bioinformatic study has analyzed associated pathways of COVID-19 by exploring genetic profiles and making correlations between ACE2 and genes related to the JAK-STAT pathway in airway epithelial cells. JAK2, STAT1, STAT2, STAT4, and STAT5A were positively correlated with ACE2 in the SARS-CoV-2 infection. However, JAK1, JAK3, STAT3, STAT5B, and STAT6 were found uncorrelated or negatively correlated [160], which is surprising due to the strong relationship of STAT3 in the pathogenesis of inflammatory and fibrotic diseases. Of note, more studies describing the molecular basis of the JAK/STAT pathway in COVID-19 are needed to address this question.

### 7.3. JAK/STAT Inhibition as Therapeutic Strategy in COVID-19

COVID-19 has proven to cause inflammation and fibrosis that can be related to the findings in ILDs. Therefore, as the JAK/STAT pathway plays an important role in the pathogenesis of ILDs, and signals the inflammatory response in COVID-19, it is not surprising that one of the therapeutic strategies under investigation for COVID-19 is blocking JAK/STAT pathway. Figure 3 shows the different JAK/STAT inhibitors that are being studied and their targets in the signaling pathway. There are currently plenty of clinical trials assessing tocilizumab in COVID-19 patients, and it has shown promising efficacy in cases with respiratory failure and ARDS [161]. However, we will not focus on treatments that do not target the JAK/STAT pathway specifically.

From all pan-JAK inhibitors, tofacitinib has no results published yet, but three ongoing clinical trials are analyzing the efficacy and safety of tofacitinib, one completed, one active, and one recruiting. There is more evidence, though, on the use of ruxolitinib and baricitinib. The first randomized controlled trial on the use of ruxolitinib in patients with severe COVID-19 showed that patients receiving this drug plus standard-of-care had better improvement and safety than the control group [162]. There are currently 19 published clinical trials with ruxolitinib in different stages of development. One of them, a phase III multicenter study, was completed (NCT04362137), which assesses the efficacy and safety of ruxolitinib in patients with COVID-19 associated cytokine storm. However, this study did not meet its primary endpoint of reducing the number of hospitalized COVID-19 patients [163]. Therefore, additional results from larger controlled studies are needed to confirm the possibility of a treatment benefit with ruxolitinib.

Concerning baricitinib, its mechanisms of action were studied. Baricitinib not only is a JAK inhibitor, but also impedes SARS-CoV-2 entrance into the target cells [158]. Therefore, some studies analyze its capability to ameliorate cytokine storm. A phase II pilot study (NCT04358614) showed an improvement of clinical characteristics and respiratory function parameters in baricitinib-treated patients, although the sample size in this trial was very low [164]. Baricitinib was also shown to reduce inflammation in ARDS patients in a preliminary multicenter study [165]. There is also a completed phase III trial (NCT04401579) with no published results, which evaluates baricitinib and remdesivir in hospitalized adult patients. The safety profile of this treatment was proven in COVID-19 patients; however, more trials are needed to evaluate its effectiveness. To date, we retrieve 15 clinical trials analyzing baricitinib for the treatment of COVID-19.

Although all evidence suggests that targeting JAK/STAT in COVID-19 might be a good strategy, it has raised a question between investigators. The inflammatory process is a natural protective response when a virus attacks the body. The triggered cytokine release is primarily regulated by JAK/STAT signaling to activate the innate immunity response. It was shown that, in stages of the disease not requiring admittance to the hospital, nearly 80% of COVID-19 patients can clear the virus through this endogenous antiviral mechanism [157,158]. Therefore, is crucial to find the appropriate target patients for the use of these drugs [166], to not impair the natural viral clearance mechanisms, and patients with a stronger cytokine storm response, appear to benefit more from the treatment with JAK/STAT inhibitors [142].

Another important question to address is the management of fibrosis derived from COVID-19 infection. In post-SARS and MERS fibrosis, IPF antifibrotic therapies, pirfenidone, and nintedanib were used [151]. However, post-inflammatory pulmonary fibrosis in COVID-19 does not have a current treatment. Pirfenidone and nintedanib were considered in an attempt to attenuate the pro-fibrotic pathways in COVID-19 patients [150,167]. In this regard, JAK/STAT inhibitors were not studied to directly ameliorate the development of fibrosis in COVID-19. However, ARDS is a risk factor for secondary pulmonary fibrosis in COVID-19 patients [151,168], and some of the clinical trials mentioned above for tofacitinib and ruxolitinib, were shown to ameliorate the cytokine storm and ARDS in COVID-19 patients. These effects show the therapeutic potential of JAK inhibitors in preventing the long-term fibrotic consequences by reducing lung injuries caused by ARDS and inflammation during COVID-19 and point them out as interesting strategies to reduce the risk factors, which will induce fibrosis in the future.

## 8. Concluding Remarks

In this review, we gathered enough evidence to conclude that JAK/STAT might play a role in the development of pathogenesis in ILDs: JAK and STAT isoforms, mostly JAK2/STAT3, were found upregulated in ILDs patients and animal models. Moreover, multiple cytokines and growth factors upregulated in ILDs activate this signaling pathway. Moreover, JAK/STAT signaling controls several cellular processes that are crucial in ILDs. Amongst them, FMT, EMT, senescence, autophagy, apoptosis, and proliferation are related to JAK/STAT activation in ILDs. However, there is still a lack of research on how this pathway takes part in the disease. Most studies focus on IPF or ILDs associated with autoimmune diseases, so much more effort is needed to completely understand the implication of JAK/STAT in other types of ILDs. This need for investigation might be the reason why there is limited clinical data about the use of JAK/STAT inhibitors in ILDs, although there are preliminary promising results in the use of JAK inhibitors, such as tofacitinib and ruxolitinib. JAK/STAT inhibitors were also proposed to ameliorate the inflammation caused by COVID-19 and the consequent fibrosis. In this regard, baricitinib and ruxolitinib are the most used. However, as well as in ILDs, there is not enough clinical data to make conclusions. Of note, all evidence arising from the efforts on COVID-19 research will help to develop strategies for ILDs, especially in those where the inflammatory state is more evident.

## Figures and Tables

**Table 1:** American Thoracic Society/European Respiratory Society classification of major idiopathic interstitial pneumonias in order of relative frequency, and their principal histologic findings and radiologic patterns.

Major Idiopathic Interstitial Pneumonias	Histologic Findings	Radiologic Pattern
Idiopathic pulmonary fibrosis (IPF)	Heterogeneous areas of patchy lung fibrosis and UIP. [3]	Basal and peripheral reticular opacities with honeycombing and traction bronchiectasis. [6]
Idiopathic nonspecific interstitial pneumonia (NSIP)	Symmetric and homogeneous UIP.	Patchy ground-glass opacities and scattered micronodules. [7]
Respiratory bronchiolitis–interstitial lung disease (RB–ILD)	Alveolar macrophages within the bronchioles.	Centrilobular nodules. Central and peripheral bronchial wall thickening. [8]
Desquamative interstitial pneumonia (DIP)	Alveolar spaces with macrophages and desquamated alveolar cells.	Extensive and diffuse ground-glass opacities with peripheral and lower lobe predominance. [3]
Cryptogenic organizing pneumonia (COP)	Tissue polyps within the alveolar ducts and alveoli, with preservation of the lung architecture.	Patchy peripheral or peribronchial consolidations predominant in the lower lung lobes and multiple nodules. [9]
Acute interstitial pneumonia (AIP)	Diffuse alveolar damage.	Extensive ground-glass opacities and areas of consolidation. [10]

Abbreviations: usual interstitial pneumonia (UIP).

**Table 2:** Summary of the different JAK/STAT signaling pathways and its activators in ILDs. The left column shows the JAK/STAT induced pathway for each activator. The right column shows the specific human ILD, ILD animal model, or cells in which the mentioned pathway was found.

JAK/STAT-Induced Pathway	ILD, ILD Animal Model and/or Cell Type
IL-4 → JAK1/JAK3 → STAT6	AECs from BLM-induced fibrosis mouse models [20,21]
IL-13 → JAK1/TYK2 → STAT6	AECs from BLM-induced fibrosis mouse model [20,21]
IL-6 → STAT3	IPF fibroblasts, normal lung fibroblasts and BLM-induced mouse model [22,23,24]
IL-11 → STAT3	BLM-induced fibrosis mouse model, human lung fibroblasts [25,26,27]
IL-31 → STAT1	Mouse pulmonary fibrosis [28]
TGF-β1 → STAT3	Human fibroblasts, AECs, SS mouse model [29,30]
TGF-β1 → JAK2 → STAT3	IPF AECs and fibroblasts, SS [31,32]
EGF → STAT3	IPF, COP, NSIP [33,34]
PDGF → STAT1	ILDs alveolar macrophages, IPF BALF [35,36,37]
FGF → STAT1/STAT3/STAT5	Progressive fibrosing ILD [38,39]
VEGF → STAT3	IPF, Idiopathic interstitial pneumonia [40,41]

Abbreviations: alveolar epithelial cells (AECs), bleomycin (BLM), systemic sclerosis (SS), idiopathic pulmonary fibrosis (IPF), cryptogenic organizing pneumonia (COP), idiopathic nonspecific interstitial pneumonia (NSIP), bronchoalveolar lavage fluid (BALF).

## Data Availability

Not applicable.
